# Diet Quality, Food Security Among Mexican-Born Women and Their Associations with Perceived Neighborhood Food Environment

**DOI:** 10.3390/nu18172857

**Published:** 2026-09-01

**Authors:** Adriana Verdezoto Alvarado, Berenice Ochoa Nogales, Juana María Meléndez Torres, Luis Huesca Reynoso, Maureen McCoy, Sonia Vega-López

**Affiliations:** 1 College of Health Solutions, Arizona State University, 500 N 3rd St, Phoenix, AZ 85004, USA; averdezoto@unm.edu (A.V.A.); bereniceochoan@gmail.com (B.O.N.); maureen.mccoy@asu.edu (M.M.); 2 Department of Individual, Family, and Community Education, University of New Mexico, Albuquerque, NM 87107, USA; 3 Coordinación de Desarrollo Regional, Centro de Investigación en Alimentación y Desarrollo, A. C., Carretera Gustavo Enrique Astiazarán Rosas No. 46, La Victoria, Hermosillo, Sonora 83304, Mexico; jmelendez@ciad.mx (J.M.M.T.); lhuesca@ciad.mx (L.H.R.)

**Keywords:** Hispanics, food security, neighborhood food environment, diet quality, immigrant Mexican women

## Abstract

**Background/Objectives**: Diet quality among Mexican-born immigrant women may be influenced by food security (FS) and neighborhood food environment factors; however, these relationships remain understudied. This exploratory study examined differences in diet quality/dietary outcomes by FS status and explored the potential for perceived neighborhood food availability to modify the association between FS and diet quality. **Methods**: Mexican-born immigrant women (*n* = 57) self-reported FS, diet, and perceived neighborhood food availability. Diet was assessed using the Southwest Food Frequency Questionnaire to calculate the Healthy Eating Index (HEI-2020) score. The six-item USDA FS Scale was used to dichotomize FS as high or low. Independent *t*-tests assessed differences in HEI-2020; fast-food frequency; and intake of fruit, vegetables, salty snacks, and sugar by FS status. An exploratory regression analysis examined whether perceived food availability modified the association between FS and diet quality using complementary Bayesian techniques to improve efficiency given the small sample size. **Results**: Women experiencing low FS had lower HEI-2020 than those with high FS (62 ± 8 vs. 66 ± 6; *p* = 0.03), as well as non-significantly lower intakes of fruit (236 ± 178 vs. 294 ± 239 g), vegetables (303 ± 188 vs. 331 ± 199 g), and salty snacks (6 ± 5 vs. 8 ± 10 g) and higher intakes of sugar (99 ± 55 vs. 96 ± 56 g) and fast food (2.5 ± 2.5 vs. 1.8 ± 1.7 times/month). No statistically significant evidence of the perceived neighborhood food availability modifying the association between FS and diet quality was observed. Exploratory analysis suggested that among women experiencing low FS, HEI-2020 score varied by perceived neighborhood food availability (low: 57; moderate: 61; high: 65; *p* = 0.077). Bayesian analyses suggested a greater impact of perceived neighborhood food availability among women with low FS and lower diet quality. **Conclusions**: In this small sample of Mexican-born women, low FS was associated with lower diet quality. Although evidence of effect modification by perceived neighborhood food availability was not observed, exploratory findings suggest that it may influence diet quality among women experiencing low FS and warrant investigation in larger studies.

## 1. Introduction

Hispanics represent a large and growing minority group in the United States (US) [1], with approximately 37.2 million individuals of Mexican origin living in the US in 2021 [2], forming the largest immigrant group in the US [3]. Mexicans and other Hispanic individuals often face adversities such as poverty, lower education and wages, and less purchasing power to obtain nutritious foods, all of which negatively impact health outcomes [4,5,6]. Low and very low food security, a condition characterized by limited access to sufficient and nutritious food [7], disproportionately affects minority populations in the US, including Hispanic households, particularly those led by women or with children [8].

In addition to individual financial challenges, lower-income communities often experience a higher density of fast-food restaurants and convenience stores [9], alongside limited access to grocery stores or supermarkets [10]. These structural disadvantages are particularly pronounced in neighborhoods with racial minority populations, where the availability of fresh, nutritious foods is restricted [11]. For individuals already experiencing food insecurity, these environmental barriers can further limit access to nutrient-dense foods, such as fruits and vegetables (F&V), while increasing exposure to energy-dense, nutrient-poor options. Together, these factors, including food insecurity, have been associated with lower diet quality [12] and highlight the challenges faced by vulnerable populations.

The built food environment plays a key role in dietary intake by influencing individuals’ food availability, accessibility, and affordability [13]. A critical component of this environment is the neighborhood food environment, which includes exposure to both healthful and unhealthful food outlets around people’s homes [14]. This environment may be characterized by objective measures and individuals’ perceptions [14]. Perceptions of the food environment have been associated with dietary intake and food consumption patterns [15,16]. Positive perceptions of the neighborhood food environment have been associated with a higher likelihood of purchasing F&V [17], while negative perceptions have been associated with increased purchases and consumption of less healthful foods [18,19].

These neighborhood food environments may be particularly relevant for immigrant populations. Immigration to the US is associated with changes in food procurement and food preparation [20]. Dietary acculturation may also influence dietary behaviors as Mexican immigrant women adapt to the host food environment after migration [21]. Immigrants, including Hispanics, have reported barriers to accessing fresh, healthy, and traditional foods [22]. For example, specialty stores such as ‘tiendas’ or ‘fruterías’, which are more common in their home countries, may offer more nutritional options than convenience stores [23]. Among Hispanic immigrant women, the presence of farmers’ markets within the home neighborhood has been associated with higher consumption of fruits, vegetables, and juice [24]. Despite these associations, limited research has examined the association between perceived neighborhood food environments and dietary behaviors among Mexican women [18], a group that plays a significant role in household food choices and family dynamics [25].

Evidence on the association between food security, neighborhood food environments, and diet quality remains inconclusive. Food insecurity and poor neighborhood food environments have each been independently associated with lower diet quality [26,27]. However, potential interactions between these factors are complex. Food security reflects economic access to food [7], whereas neighborhood food environments reflect physical access to healthy foods [13]. Unlike objective measures of the food environment, perceived food availability captures individuals’ experiences of food accessibility, affordability, and quality, which may shape purchasing and dietary behaviors. Accordingly, perceptions of the neighborhood food environment among immigrant populations may be shaped by language barriers, transportation, acculturation, and cultural food preferences [22]. Therefore, research examining perceived neighborhood food environments as a potential modifier of the relationship between food security and diet quality among immigrants is warranted.

Hence, this study aimed to compare diet quality and dietary outcomes between Mexican-born women with high and low food security and to explore whether the perceived neighborhood food environment may modify the relationship between food security and diet quality in this population. We hypothesized that women with lower food security would have less favorable diet quality than those with higher food security and that the perceived neighborhood food environment would modify this association such that the relationship between food security and diet quality would differ depending on perceived neighborhood food availability.

## 2. Materials and Methods

This secondary data analysis was part of a larger study exploring sociocultural factors contributing to chronic disease risk among Mexican-born women living in the Southwestern US [28]. The current analysis focused on food security, diet quality, and perceived neighborhood food environment as individual and contextual factors influencing dietary behaviors. Cross-sectional data were collected via an interviewer-administered paper survey from February to August 2019. Mexican-born women (25–50 years; *n* = 61) residing in the US for at least 12 months who were either employed full-time or a full-time homemaker participated in this study. Women with part-time employment were excluded from the parent study to reduce variability in the time potentially available for diet-related behaviors and exposure to the neighborhood food environment. As defined by the parent study, exclusion criteria were being pregnant/breastfeeding; known chronic diseases (e.g., diabetes mellitus, active cancer); restrictive diet (e.g., very low carbohydrate diets), medications likely to impact metabolic outcomes (e.g., metformin); and weight change (±5 kg) in the prior 6 months. Recruitment occurred through announcements in meetings and flyers posted in churches, community centers, community-based organizations, and through word of mouth. Once a potential participant expressed interest in partaking in this study, they provided verbal consent to complete a screening questionnaire, administered in person or via phone, to verify eligibility. Women self-reported their birth country during the screening process. After explaining the study in detail, written informed consent was obtained prior to conducting any study-related activities. Participants provided written informed consent (English or Spanish) and were compensated for their time via a $30 gift card. This study was conducted in accordance with the Declaration of Helsinki and approved by the Institutional Review Board at Arizona State University.

### 2.1. Data Collection

As part of this study, participants attended a one-time visit at the Arizona Biomedical Collaborative research facilities. During this visit, they completed a survey with the assistance of trained bilingual interviewers, who verbally administered the questions in the participant’s preferred language (English or Spanish) and recorded their responses on a paper survey. The survey collected sociodemographic information, food security status, information about their perceived neighborhood food environment, and dietary intake data. Validated Spanish versions of the questions were used when available. If needed, questions were translated into Spanish, and the translation was verified by a bilingual researcher unaffiliated with the project. Completing the survey required approximately 90 to 120 min.

### 2.2. Measures

Sociodemographic information collected included age, marital status, monthly household income, level of education completed, time in the US, and current employment status.

Food security was assessed using the US Department of Agriculture short-form six-item food security survey module, which assessed food security over the previous 12 months [7]. After adding raw scores, participants who scored 0–1 were placed in the high or marginal food security scale, participants with scores of 2–4 were considered to have low food security, and participants with scores of 5–6 were classified as having very low food security. Food security status was dichotomized prior to conducting statistical analyses. Participants with high or marginal food security were placed in the high-food-security group, and participants with low or very low food security were placed in the low-food-security group.

The perceptions of the neighborhood food environment scale are a reliable measure [29] of perceived availability and accessibility to food outlets (20 min walk or 1-mile from home). The scale asks the following questions: “A large selection of fresh F&V is available in my neighborhood”, “The fresh F&V in my neighborhood are of high quality”, and “A large selection of low-fat products is available in my neighborhood.” Responses range from 1 = strongly agree to 5 = strongly disagree and are summed (3–15 range), with higher values indicating less availability of F&V and low-fat products. In the present sample, the scale demonstrated acceptable internal consistency (Cronbach’s α = 0.82). For this analysis, final scores were categorized into tertiles based on the distribution of scores in the present sample to facilitate comparisons across the levels of perceived neighborhood availability. Scores of 3 to 5.81 for the three questions were classified as high availability, scores of 5.82 to 8 as medium availability, and scores of 8.01 to 15 as low availability.

Dietary intake was assessed using the Southwest Food Frequency Questionnaire (SWFFQ), a semi-quantitative 175-item food frequency questionnaire, which asks respondents to report frequency of consumption (captured as number of times per day, week, or month) of each food item over the prior 12-month period. Respondents also indicate whether their usual portion size of the food is small, medium, or large. Age–gender-specific portion size assumptions are used in the calculations of intake estimates of nutrients, foods, and food groups. The SWFFQ has been tested for reliability and validity for the Hispanic population [30]. SWFFQ data were used to estimate total fruit, vegetable, and sugar consumption and intake of salty snacks, and they were used to calculate the Healthy Eating Index (HEI)-2020 total score [31] using the procedures outlined by the National Cancer Institute [32]. In brief, the HEI-2020 total score indicates compliance with the Dietary Guidelines for Americans and is scored from 0 to 100, with higher scores indicating greater adherence [31]. The adequacy and moderation components contributing to the total HEI-2020 score have been described elsewhere [33].

### 2.3. Statistical Analyses

Of the 61 women enrolled in this study, one was excluded due to missing dietary data. Three participants had an overall reported energy intake greater than three standard deviations from the mean, which was deemed implausible, and therefore, they were excluded from the analysis. A total of 57 women were included in the present analysis.

All variables were checked for violations of normality; intake of salty snacks was natural log-transformed to achieve normality prior to conducting statistical analysis. Descriptive statistics are presented as mean (standard deviation) for continuous variables and frequency with percentages for categorical values. Independent samples *t*-tests were conducted to explore the mean differences in diet outcomes between participants with high food security and those with low food security.

Unadjusted linear regression models evaluated associations between demographic variables and diet quality to determine covariates in the model based on at least some evidence of a statistically significant relationship (*p* < 0.1). Based on these criteria, no covariates were controlled for in the exploratory moderation analyses. An exploratory moderation analysis was carried out using the PROCESS macro for SPSS [34] to assess whether the perception of F&V and low-fat availability may modify the relationship between food security and diet quality. The PROCESS macro conducts linear regression analysis with an interaction term to test moderation hypotheses [35]. Samples were centered and bootstrapped (5000 resamples) in all moderation models. To avoid errors, we employed the heteroscedasticity HC3 model (Davidson–MacKinnon), which has been recommended for small sample sizes and when heteroscedasticity may be present [36]. The PROCESS macro created an interaction term to test the effect of the moderator and the independent variable on the model. Constant values and coefficients, such as beta values, standard error, t- and *p*-values, are presented in the Section 3 for an easier construct of the full regression model. A Bayesian seemingly unrelated bivariate probit model (SUR-biprobit) was estimated as a complementary analysis for the two endogenous binary variables, FS and HEI. This framework jointly estimates both equations while allowing for correlated latent disturbances, enabling quantification of the joint probabilities of the four possible outcome combinations and assessment of whether unobserved factors simultaneously influence FS and HEI after controlling for perceived neighborhood food availability and other relevant sociodemographic covariates (Supplementary Materials S1). Statistical analyses were carried out using the Statistical Program for Social Sciences (SPSS, Version 24; IBM Corp., Armonk, NY, USA) and Stata for the Bayesian analysis (Version 18, Stata Corp LLC, College Station, TX, USA).

## 3. Results

Overall, participants with low food security had significantly lower diet quality compared with those with high food security. Exploratory analyses suggested that perceived neighborhood food availability may be related to differences in diet quality among participants experiencing low food security, although no significant interaction was observed. Table 1 summarizes sample characteristics. Participants were 41 ± 7 years (range of 25 to 50 years old) with a mean time in the US of 20 ± 9 years (range 1 to 43 years). The mean perceived neighborhood availability of F&V and low-fat products score was 6.9 ± 3.2. Mean gram consumption was 278 ± 224 for fruits, 323 ± 194 for vegetables, 97 ± 56 for total sugar, and 7 ± 9 for salty snacks. Mean fast-food consumption frequency was 2 ± 2 times/month. The mean HEI-2020 total score was 64.4 ± 6.6.

Participants with low food security had a significantly lower HEI-2020 score (61.5 ± 8.2) than those with high food security (65.6± 5.6; 95% CI [0.29, 7.87]; *p* = 0.035; Cohen’s *d* = 0.64). Participants with low food security also reported non-significantly lower fruit intake (236 ± 178 vs. 294 ± 239 g; 95% CI [−74.8, 189.91]; *p* = 0.39; *d* = 0.26), vegetable intake (303 ± 188 vs. 331 ± 199 g; 95% CI [−87.94, 143.11]; *p* = 0.634; *d* = 0.14), and salty snack intake (6 ± 5 vs. 8 ± 10 g; 95% CI [−2.85, 7.76]; *p* = 0.357; *d* = 0.29) compared to those with high food security. In contrast, individuals with low food security reported non-significantly higher sugar intake (99 ± 55 vs. 96 ± 56 g; 95% CI [−35.4, 30.8]; *p =* 0.890; *d* = 0.04) and fast-food consumption (2.5 ± 2.5 vs. 1.8 ± 1.7 times/month; 95% CI [−2.04, 0.82]; *p* = 0.145; *d* = 0.31).

Table 2 shows the results from exploratory moderation analyses examining whether perceived F&V and low-fat product availability scores were associated with diet quality and whether they modified the relationship between FS status and HEI−2020. The overall model explained a small proportion of variance in HEI-2020 (R^2^ = 0.154, F(3, 52) = 2.39, *p* = 0.077).

**Figure 1:**
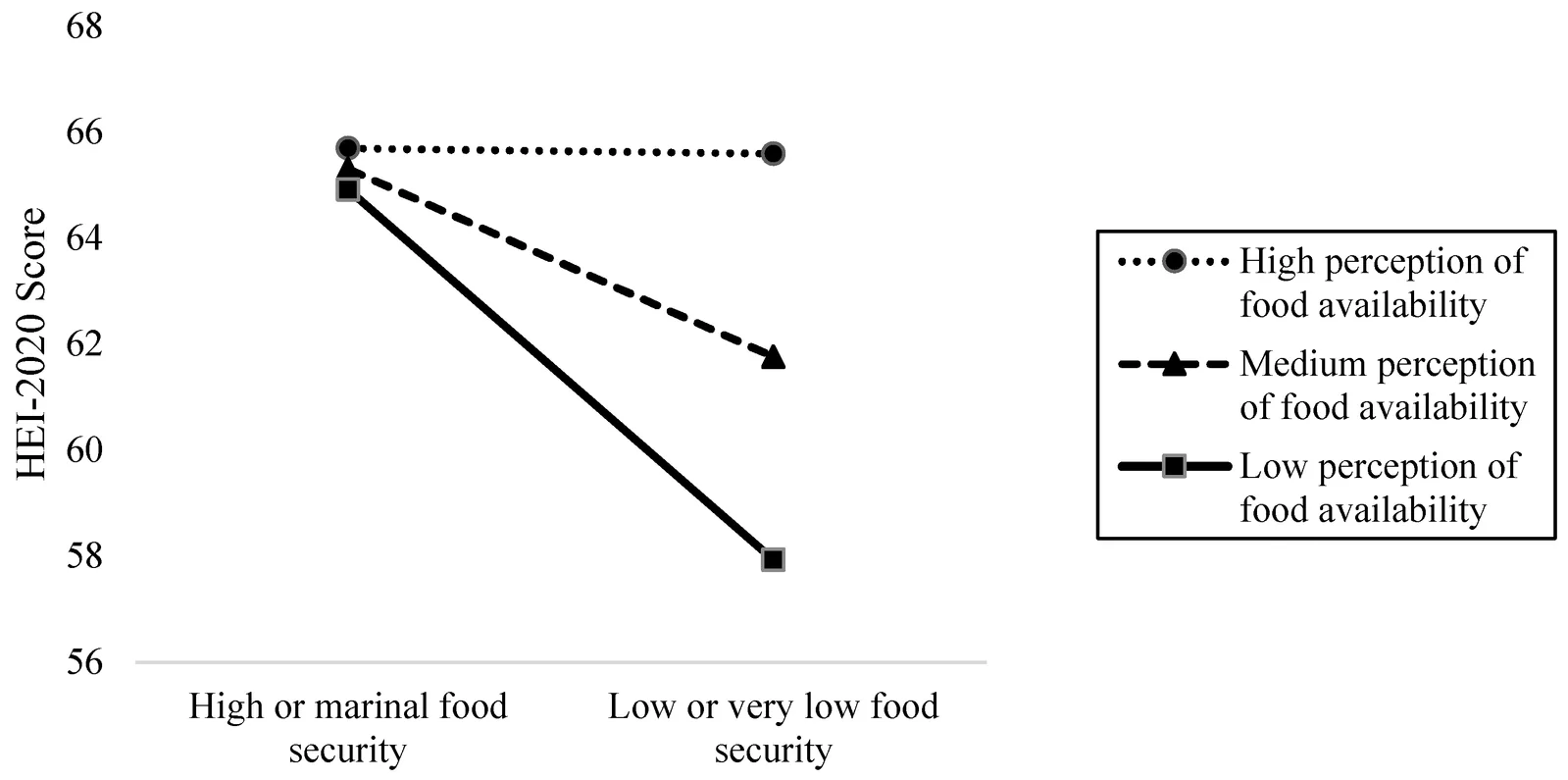
Simple slope equations of the regression of HEI-2020 on food security at three levels of perceptions of fruit, vegetable, and low-fat availability. R^2^ = 0.154 and *p* = 0.077. Subgroup (*n* = 56) sample sizes were as follows: high food security/high perceived availability (*n* = 14), high food security/medium perceived availability (*n* = 13), high food security/low perceived availability (*n* = 13), low food security/high perceived availability (*n* = 4), low food security/medium perceived availability (*n* = 7), and low food security/low perceived availability (*n* = 5).

Figure 1 illustrates differences between food security and HEI-2020 score based on the perceived neighborhood food environment. HEI-2020 total scores were relatively stable among participants with high food security across varying perceptions of food availability (65.7 for high, 65.3 for medium, and 64.9 for low). Among participants with low food security, HEI-2020 total scores appeared lower among those reporting low perceived neighborhood food availability (57.9) compared to those reporting medium (61.7) or high (65.6) perceived availability.

The results from the Bayesian analyses are included in Supplementary Materials S1. The relevant findings reveal that with longer time in the US, perceived food availability is less relevant among women with high food security and greater diet quality, whereas it slightly increases the probability of having greater diet quality among women with low food security.

## 4. Discussion

This study examined differences in diet quality and dietary outcomes among Mexican-born women with differing levels of food security and explored whether the perceived neighborhood food environment may modify the association between food security and diet quality. This study contributes to the limited literature examining how perceived neighborhood food environments may shape the relationship between food security and diet quality among Mexican-born immigrant women, a population susceptible to social and environmental barriers to healthy eating [37]. Consistent with previous research [38], individuals from households with low food security had lower HEI-2020 scores compared to their food secure counterparts. Despite the difference in HEI-2020 scores being small, the moderate effect size suggests potential meaningful differences in overall diet quality, underscoring the importance of addressing food security as an important factor associated with diet quality in this population.

To further contextualize differences in the HEI-2020 total score, dietary components were also examined. Existing evidence supports the association between food insecurity and F&V intake [9]. Although not statistically significant, study findings indicated that individuals from households with high food security reported higher F&V intake and lower sugar and fast-food consumption compared to those from households with low food security. These differences in dietary components follow the same direction as observed differences in HEI-2020 total scores and may reflect underlying dietary behaviors contributing to overall diet quality [28].

Low food security has consistently been associated with lower dietary quality, and neighborhood food environment factors such as fast-food outlet proximity have been proposed as a potential moderator of this association in prior research [39]. Our exploratory findings indicated that women with low food security and a low perception of food availability in their neighborhoods tended to have a lower HEI-2020 score (*p* = 0.077). Specifically, among women with low food security, those with a high perception of food availability (65.6) had an HEI-2020 score that was approximately 7.7 points higher compared to those with low perception of food availability (57.9). However, this difference in HEI-2020 total scores was not observed among women with high food security, where those with a high perception of food availability (65.7) had only a slightly higher score than those with a low perception (64.9). Albeit relatively small in magnitude, the difference in HEI-2020 scores may be of public health relevance, as a previous review suggested that a 5- to 6-point improvement in diet quality scores may represent meaningful changes in dietary patterns to reduce cardiometabolic risk [40]. The complementary Bayesian analysis suggested that, with longer US residence, perceived food availability was less relevant for the probability of having both high food security and a healthy diet. These findings should be interpreted in light of the cross-sectional and exploratory nature of this study and the complex interplay of factors contributing to dietary behaviors. Nevertheless, the observed differences in diet quality by perceived neighborhood food availability may modify the association between food security and diet quality. Further fully powered studies are warranted to confirm this potential modifying effect.

The mechanisms through which food insecurity influences diet quality are complex. Food insecurity is a social determinant of health that is intertwined with broader indicators of financial instability and overall social risk [41,42]. Individuals who experience food insecurity often experience other barriers to accessing and procuring healthy food options, such as lack of transportation, competing responsibilities, time constraints, and psychological stress [43,44]. The varying levels of education and income within our sample further underscore the influence that socioeconomic factors might have on dietary habits and access to healthy foods. The perception of low food availability may act as a barrier, potentially exacerbating the challenges women with food insecurity face and hindering their ability to make healthier food choices. This aligns with findings from the Hispanic Community Health Study, which suggest that perceptions of the neighborhood food environment could be considered in structural neighborhood environment interventions [45]. These findings support the use of ecological frameworks [46] to further examine how food insecurity and perceived neighborhood food environments may shape dietary behaviors. Furthermore, the possibility that sociocultural traits common among immigrant Mexican women may play a role in the relationship between food insecurity and diet quality cannot be ruled out [20,22], highlighting the need to develop public health interventions that better address the socioeconomic and cultural needs of diverse populations and improve access to nutritious foods [11].

While this study contributes insights into a population that plays a pivotal role in household food choices and family dynamics [25], it is not without limitations. Data were self-reported, which could have resulted in biases in reported dietary intake and perceptions. Diet assessment via food frequency questionnaires is known to pose important reporting challenges. Most participants (98%) responded to the survey in Spanish, reflecting a specific linguistic and cultural context and reducing the likelihood that language differences may have influenced participant responses. Thus, findings might not be applicable to all Hispanics or even to all Mexican-born populations in the US. Moreover, eligibility criteria for this secondary analysis were based on the parent study; restricting eligibility to full-time employment or full-time homemakers may limit the generalizability of findings to women in other employment situations. Recruitment strategies (e.g., word of mouth) used in this study may have contributed to selection bias, resulting in a sample that is not representative of all Mexican-born women living in this region. The neighborhood food environment perception scale is subjective and may be influenced by participants’ perceived distance and awareness of food availability, which may not fully capture the complexity of environmental factors influencing dietary behaviors. The relatively small sample size, particularly the number of participants with low food security, may have limited the statistical power to detect significant differences for some dietary outcomes. Although several dietary variables showed consistent numerical differences between groups, these findings should be interpreted with caution, as the possibility of Type II errors cannot be excluded. Sensitivity analyses excluding participants with implausible energy intake were not conducted in the current analysis. The complementary Bayesian analysis allowed estimations of joint probabilities of food security and diet quality across levels of perceived food availability; however, these estimates remain exploratory given the small sample and cross-sectional design. Future studies including larger and more diverse samples will help establish the generalizability of these findings. Finally, a cross-sectional analysis does not allow for inferring causality or determining the directionality of the associations tested.

## 5. Conclusions

This study examined whether the perceived neighborhood food environment was associated with diet quality/dietary outcomes and whether it may modify the association between food security and diet quality among Mexican-born women residing in a metropolitan area in the Southwestern US. The main study findings corroborate an association between food security and diet quality. The results analysis regarding the potential role of neighborhood food environment perceptions as a factor that contributes to this association should be interpreted cautiously given the small sample size. Further research with larger samples and more robust observational designs is needed to confirm these associations and to further explore the role of the neighborhood food environment as a contextual factor that impacts diet quality. Additional research to understand how the perceived and objectively measured neighborhood food environment influences diet quality is warranted to better understand how environmental and socioeconomic factors may jointly contribute to dietary behaviors in this population. Future findings can inform public health strategies, including those involving nutrition assistance programs and community-based interventions and policies aimed at improving food security, diet quality, and access to nutritious foods among Mexican-born immigrant women.

## Figures and Tables

**Table 1:** Sample characteristics of study participants (*n* = 57).

	*N*	%
Marital status		
Married and living with spouse	26	45.6
Married but not living with spouse	4	7.0
Not married but living with partner	10	17.5
Single	11	19.3
Divorced	6	10.5
Educational attainment		
Less than high school	26	45.6
Complete high school but incomplete college	15	26.3
College graduate or higher	16	28.1
Household monthly income		
<$2000	24	42.1
$2000–4000	18	31.6
>$4000	11	19.3
Did not know/not sure	4	7.0
Employment status		
Working full-time (40 h or more per week)	32	56.1
Homemaker	25	43.9
Food security		
High food security	41	71.9
Low food security	16	28.1
Language spoken at interview		
Spanish	56	98
English	1	2
Availability of fruits, vegetables, and low-fat products ^1^		
High perception	18	31.6
Medium perception	20	35.1
Low perception	18	31.6

^1^ *n* = 56.

**Table 2:** Exploratory linear regression models examining perceived fruits, vegetables, and low-fat product availability as a potential moderator of the association between food security and diet quality (*n* = 56).

	b (SE)	95% CI	T	*p*
HEI-2020				
Constant	64.32 (0.86)	[62.60, 66.04]	75.14	0.000
FS Categories	−3.47 (2.20)	[−7.89, 0.94]	−1.58	0.119
AFVL	−0.33 (0.25)	[−0.84, 0.17]	−1.32	0.191
FS × AFVL	−1.12 (0.62)	[−2.37, 0.12]	−1.81	0.077

Abbreviations: AFVL, availability of fruits, vegetables, and low-fat products; FS, food security. Note. Confidence intervals and standard errors were estimated using 5000 bootstrap samples.

## Data Availability

The raw data underlying the conclusions of this article are available from the authors upon request.

## Supplementary Materials

The following supporting information can be downloaded at https://www.mdpi.com/article/10.3390/nu18172857/s1. Supplementary Materials S1: Methodology and results from the complementary Bayesian analysis exploring the role of perceived neighborhood food availability on the association between diet quality and food security. Figure S1. Joint probabilities of healthy diet and household food security by perceived food availability. Table S1. Bayesian posterior estimates of the latent error correlation. Table S2. Estimates of probit models and Bayesian seemingly unrelated bivariate probit models (SUR-biprobit).

## Abbreviations

The following abbreviations are used in this manuscript:

FS	Food Security
F&V	Fruit and Vegetable
SWFFQ	Southwest Food Frequency Questionnaire
