# Supplementary material for: Diet Quality, Food Security Among Mexican-Born Women and Their Associations with Perceived Neighborhood Food Environment
Source: Nutrients. 2026 Sep 1;18(17):2857. doi: 10.3390/nu18172857 (PMC13567579; doi:10.3390/nu18172857)
Supplement: Supplementary file 1 [file nutrients-18-02857-s001.zip › nutrients-4471384-supplementary.pdf]

Diet Quality, Food Security among Mexican-born Women and their Associations with Perceived Neighborhood Food Environment

Verdezoto Alvarado, et al.

Supplementary Materials S1. Methodology and results from the complementary Bayesian analysis exploring the role of perceived neighborhood food availability on the association between diet quality and food security.

Statistical Analysis

As a complementary analysis, we estimated a Bayesian seemingly unrelated bivariate probit model (SUR-biprobit) for the two endogenous binary variables, FS and healthy diet quality (HEI). This framework jointly estimates both equations while allowing for correlated latent disturbances, enabling quantification of the joint probabilities of the four possible outcome combinations and assessment of whether unobserved factors simultaneously influence FS and HEI after controlling for perceived neighborhood food availability (FV) and other relevant sociodemographic covariates. Bayesian estimation with weakly informative priors and Markov Chain Monte Carlo (MCMC) sampling provides a flexible inferential framework that is particularly advantageous in relatively small samples because it fully characterizes posterior uncertainty without relying exclusively on large sample asymptotic approximations. This latter approach is novel, as it will run over a Bayesian flow of parameters regardless of the small number of observations in our database. This analysis was carried out using Stata (Version 18, College Station: Stata Corp.).

The model is estimated using MCMC with 20,000 posterior draws and weakly informative priors. Bayesian modeling in this phase jointly estimates the latent error correlation (Athrho) through MCMC sampling due to the small sample. This parameter is central to the analysis because it quantifies whether unobserved factors simultaneously influence HEI and FS beyond the observed socioeconomic characteristics. Therefore, inference on Athrho provides a direct assessment of the appropriateness of the joint model relative to estimating two independent regressions.

Results

Figure S1 illustrates differences between food security and healthy diet scores based on the perceived neighborhood food environment. The four joint probabilities follow different trajectories for each of the perceived food availability (FV) in the neighborhood. The likelihood of simultaneously having an unhealthy diet and low FS (Pr[HEI=1 ; HFS = 1]) increases during the first year of U.S. residence, reaching a peak around 30 years before slightly declining. In

contrast, the odds of an unhealthy diet with high FS (Pr[HEI=1 ; HFS = 0]) decrease steadily as residence length increases. Conversely, the probability of a healthy diet despite low FS (Pr[HEI=0 ; HFS = 1]) rises continuously over time, whereas the joint probability of a healthy diet and high FS (Pr[HEI=0 ; HFS = 0]) remains relatively stable with only modest variations. Joint probabilities on these four schemes' graphs are additive and add to the value of one as a result of the additive property of the joint estimation.

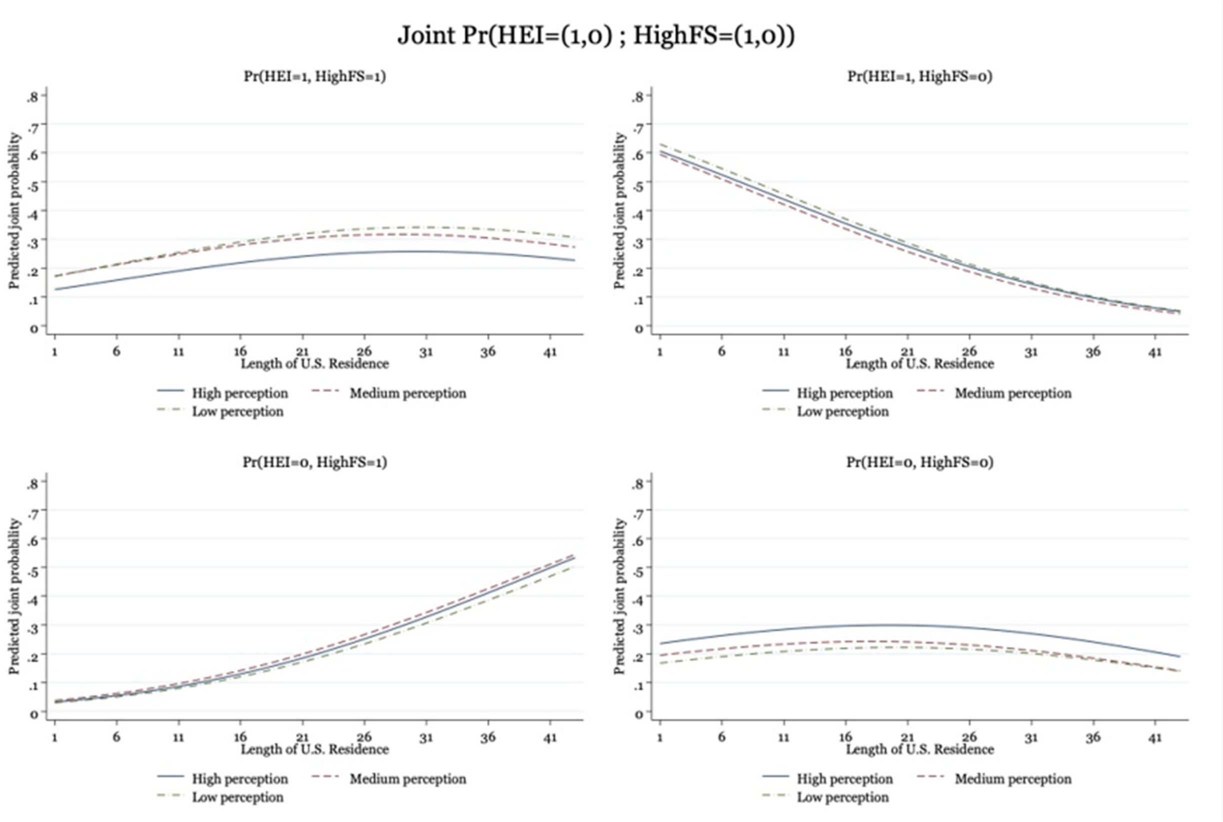

Figure S1. Joint probabilities of healthy diet and household food security by perceived food availability. Longer U.S. residence is associated with lower probabilities of a healthy diet alone and higher probabilities of food security alone, whereas the joint probability of a healthy diet and food security changes only modestly across perception levels.

Table S1 presents the Bayesian test for a positive correlation between the two specifications, using the parameter rho and its corresponding posterior estimate. The posterior mean of the latent error correlation (art-rho) was 0.139, with a 95% c.i. ranging from -0.286 to 0.591, indicating some uncertainty regarding the direction and magnitude of the residual dependence between the two probit equations. Likewise, the posterior distribution of Athrho was centered

close to zero (mean = 0.132; 95% c.i.: -0.278 to 0.531). As both c.i. values include the value of zero within the range, the Bayesian evidence suggests little residual correlation between the latent equations after controlling for the attributes considered. Therefore, joint estimation allows for potential dependence between equations; although the posterior results indicate that such dependence is weak, it still plays an important role by providing direct estimates of the four outcome probabilities, offering richer policy insights than separate probit models.

Table S1. Bayesian posterior estimates of the latent error correlation.

Parameter	Posterior estimate and C.I.
ρ = tanh (*Athrho*)	Mean = 0.139; SD= 0.223; MCSE= 0.0047; Median= 0.134; 95% c.i.= (-0.286, 0.591)
Athrho (Fisher's transformation of rho)	Mean = 0.132; SD= 0.208; MCSE= 0.0044; Median= 0.134; 95% c.i.= (-0.278, 0.531)

Note: Bayesian seemingly unrelated bivariate probit with 20,000 Monte Carlo draws.

Formalization and Estimates

A Bayesian seemingly unrelated bivariate probit model is implemented for the two binary categories of healthy eating index (HEI) and food security (FS), which simultaneously estimate the following system with a vector X_i of covariates:

$$HEI_i^* = X_i\beta_{i1} + \varepsilon_{i1}$$

$$FS_i^* = X_i\beta_{i2} + \varepsilon_{i2}$$

We allow

$$Cov^*(\varepsilon_1, \varepsilon_2) = \rho$$

with

$$(\varepsilon_1, \varepsilon_2) \sim N\left(\begin{bmatrix}0\\0\end{bmatrix}, \begin{bmatrix}1 & \rho\\ \rho & 1\end{bmatrix}\right)$$

We define

$$\eta_1 = X_1\beta_1$$

$$\eta_2 = X_2\beta_2$$

Considering the bivariate normal cumulative distribution function (CDF) Φ_2 , the probability of both events occurring is

$$\mathrm{P}_{11} = \mathrm{P}(y_1 = 1 , y_2 = 1) = \Phi_2(\eta_1, \eta_2; \rho)$$

In our case, each case would be, respectively,

$$\mathrm{P}(HEI = 1 , FS = 1).$$

If the first event yields yes and the second yields no,

$$\mathrm{P}_{10} = \mathrm{P}(y_1 = 1 , y_2 = 0) = \Phi_2(\eta_1, -\eta_2; -\rho)$$

If the first event yields no and the second yields yes,

$$\mathrm{P}_{01} = \mathrm{P}(y_1 = 0 , y_2 = 1) = \Phi_2(-\eta_1, \eta_2; -\rho)$$

If neither events yield yes,

$$\mathrm{P}_{00} = \mathrm{P}(y_1 = 0 , y_2 = 0) = \Phi_2(-\eta_1, -\eta_2; \rho)$$

And the final property that all events add up to one is

$$P_{11} + P_{10} + P_{01} + P_{00} = 1.$$

Considering the sample with Markov Chain Monte Carlo simulations, we formally obtain

$$P_{11}^{(m)} = \Phi_2\left(X_1\beta_1^{(m)}, X_2\beta_2^{(m)}; \rho^{(m)}\right)$$

where $m = 1, \dots, 20000$. Generally, the following probabilities with the Bayesian expression are

$$P(y_1 = i , y_2 = j \mid X) = \Phi_2\left(s_i X_1 \beta_1, s_j X_2 \beta_2; s_i s_j \rho\right), \quad i,j \in (0 , 1),$$

where

$$s_i = \begin{cases} 1, & i = 1 \\ -1, & i = 0 \end{cases} \qquad \& \qquad s_j = \begin{cases} 1, & j = 1 \\ -1, & j = 0 \end{cases}$$

Table S2. Estimates of probit models and Bayesian seemingly unrelated bivariate probit models (SUR-biprobit).

	Bayes:SUR-Biprobit				Bayes:SUR-Biprobit			
	Healthy diet (HEI)				Food security			
	Mean	MCSE[1]	Equal-tailed [95% C.I]		Mean	MCSE[1]	Equal-tailed [95% C.I]	
			Lower	Upper			Lower	Upper
Age	0.172	(0.013)	0.027	0.321	0.100	(0.003)	-0.007	0.207
Age squared	-0.002	(0.000)	-0.004	0.001	-0.002	(0.000)	-0.004	0.000
Length of residence	-0.037	(0.002)	-0.088	0.016	0.068	(0.005)	-0.001	0.140
Married	-0.122	(0.021)	-0.731	0.462	0.170	(0.035)	-0.513	0.850
Labor status	0.451	(0.026)	-0.247	1.116	-0.605	(0.051)	-1.356	0.219
Schooling								
High school	Base				Base			
but incomplete college	-0.862	(0.043)	-1.527	-0.193	0.071	(0.052)	-0.535	0.658
College or higher	-0.945	(0.026)	-1.724	-0.198	-0.584	(0.118)	-1.991	0.703
incomegroups								
< $2,000	Base				Base			
$2,000-$4,000	0.392	(0.032)	-0.307	1.086	-0.871	(0.032)	-1.304	-0.423
> $4,000	0.301	(0.046)	-0.464	1.089	-1.308	(0.039)	-2.233	-0.498
Did not know/refuse	-1.635	(0.048)	-2.565	-0.790	-0.221	(0.150)	-2.019	1.371
FV neighborhood								
High availability	Base				Base			
Medium availability	0.106	(0.014)	-0.643	0.865	0.257	(0.037)	-0.654	1.044
Low availability	0.366	(0.062)	-0.351	1.092	0.308	(0.050)	-0.575	1.210
Constant	-3.351	(0.150)	-5.263	-1.524	-1.588	(0.026)	-2.593	-0.604
Athrho (posterior)	0.139	(0.005)	-0.286	0.591				
Observations	57							
MCMC sample size	20,000							
Athrho (posterior)		-0.139***					-0.132***	
MCSE/[1]		(0.005)					(0.004)	

**** MCSE should be less than about 5% of the posterior SD.*

[1/] Monte Carlo Standard Error between parentheses.

Note: Specifications include age squared and the same group of covariates in both equations.
